# Clinical characterization of two severe cases of hemorrhagic fever with renal syndrome (HFRS) caused by hantaviruses Puumala and Dobrava-Belgrade genotype Sochi

**DOI:** 10.1186/s12879-016-2012-2

**Published:** 2016-11-14

**Authors:** Ellen Krautkrämer, Christian Nusshag, Alexandra Baumann, Julia Schäfer, Jörg Hofmann, Paul Schnitzler, Boris Klempa, Peter T. Witkowski, Detlev H. Krüger, Martin Zeier

**Affiliations:** 1 Department of Nephrology, University of Heidelberg, Im Neuenheimer Feld 162, Heidelberg, 69120 Germany; 2 Institute of Medical Virology, Charité Medical School, Berlin, Germany; 3 Department of Virology, University of Heidelberg, Heidelberg, Germany; 4 Institute of Virology, Biomedical Research Center, Slovak Academy of Sciences, Bratislava, Slovakia

**Keywords:** Hantavirus, Dobrava-Belgrade virus genotype Sochi, Puumala virus, clinical severity, cytokines

## Abstract

**Background:**

Hantavirus disease belongs to the emerging infections. The clinical picture and severity of infections differ between hantavirus species and may even vary between hantavirus genotypes. The mechanisms that lead to the broad variance of severity in infected patients are not completely understood. Host- and virus-specific factors are considered.

**Case presentation:**

We analyzed severe cases of hantavirus disease in two young women. The first case was caused by Puumala virus (PUUV) infection in Germany; the second case describes the infection with Dobrava-Belgrade virus (DOBV) in Russia. Symptoms, laboratory parameters and cytokine levels were analyzed and compared between the two patients. Serological and sequence analysis revealed that PUUV was the infecting agent for the German patient and the infection of the Russian patient was caused by Dobrava-Belgrade virus genotype Sochi (DOBV-Sochi). The symptoms in the initial phase of the diseases did not differ noticeably between both patients. However, deterioration of laboratory parameter values was prolonged and stronger in DOBV-Sochi than in PUUV infection. Circulating endothelial progenitor cells (cEPCs), known to be responsible for endothelial repair, were mobilized in both infections. Striking differences were observed in the temporal course and level of cytokine upregulation. Levels of angiopoietin-2 (Ang-2), vascular endothelial growth factor (VEGF), and stromal derived factor-1 (SDF-1α) were increased in both infections; but, sustained and more pronounced elevation was observed in DOBV-Sochi infection.

**Conclusions:**

Severe hantavirus disease caused by different hantavirus species did not differ in the general symptoms and clinical characteristics. However, we observed a prolonged clinical course and a late and enhanced mobilization of cytokines in DOBV-Sochi infection. The differences in cytokine deregulation may contribute to the observed variation in the clinical course.

## Background

Diseases caused by hantaviruses differ enormously in severity and clinical course. Host- and virus-specific determinants are discussed as reasons for the broad range of clinical pictures [1–5]. The most obvious differences exist between the clinical picture of hantaviral cardiopulmonary syndrome (HCPS) and HFRS caused by New and Old World hantaviruses, respectively [6]. Whereas HCPS manifests predominantly in the lung, HFRS is mostly characterized by renal failure. However, there is also a broad variety of symptoms in hantavirus disease caused by Old World hantaviruses. In contrast to Hantaan virus (HTNV), infection with PUUV is associated in most cases with a mild form of HFRS. Various genotypes exist within the species Dobrava-Belgrade virus and they cause diseases of different severity [7]. In addition, hantavirus infection exhibits individual differences ranging from subclinical to fatal outcome.

The reasons for the variation of severity between virus species/genotypes and in individual patients are not yet known. Diverse determinants concerning virus- and patient-specific characteristics may play a role in the pathogenesis. Differences in the use of entry receptors, in the regulation of cytokine response and in viral replication were described to be associated with pathogenicity [8–11]. Studies with genetic reassortants in vitro and in animal models suggest molecular determinants to be responsible for virulence [5, 12]. However, the species-specific factors of hantaviruses that are responsible for pathogenicity and clinical picture are not identified so far. Interestingly, the pathogenicity of related viruses of DOBV genotypes differs enormously with case fatality rates (CFRs) between 0.3%-0.9% for DOBV genotype Kurkino and 14.5% for DOBV genotype Sochi [13]. In addition to severe courses that are linked to specific virus species or genotypes, several serious cases were reported for infection with PUUV that usually causes a milder form of hantavirus disease [14, 15]. These infections often involve extrarenal manifestations [16, 17]. Severe cases caused by various hantavirus species are not well characterized with regard to their differences and similarities in symptoms, organ involvement, laboratory parameters, and clinical course.

The comparison of the clinical picture and course of severe disease caused by different hantavirus species may provide useful insights into their pathogenicity. Therefore, we analyzed the course of two severe hantavirus cases caused by infection with PUUV and DOBV-Sochi.

## Case presentation

We report on hantavirus disease of a 25-year old German and a 20-year-old Russian woman infected with hantavirus PUUV and DOBV, respectively. Patients infected with hantavirus PUUV and DOBV were hospitalized in the Department of Nephrology, University of Heidelberg, Germany, in 2012 and 2014, respectively. The infection with DOBV occurred in the district of Krasnodar, South Russia, and the one with PUUV in Heidelberg, Germany. Infection was diagnosed by positive IgG and IgM hantaviral serology (*recom*Line HantaPlus assay, Mikrogen Diagnostik). Admission was on day four and on day six after onset of symptoms for the patient with PUUV and DOBV infection, respectively. To analyze the genotype of DOBV and to obtain partial nucleotide sequences of genomic segments, RT-PCR of serum and urine samples with subsequent sequencing was performed as described previously [18, 19]. Hantaviral RNA was detected in serum but not in urine. Partial nucleotide sequences of S, M, and L segments amplified from serum derived from the DOBV-Sochi patient were deposited in GenBank (accession numbers KU529946, KU529944 and KU529945). The sequences showed high similarity to DOBV-Sochi sequences obtained from Black Sea field mice (*Apodemus ponticus*) and from a fatal case of hantavirus disease reported in a 47-year-old woman in the district of Krasnodar in southern European Russia (Table 1) [13, 20, 21]. No pre-existing conditions, such as renal, pulmonary or cardiovascular disease, diabetes mellitus, hypertension or obesity, were found. Body weight and height were similar between the patients. Both patients were non-smokers. Symptoms during the early phase of both cases were very similar (Table 2). Both cases showed the typical initial signs of hantavirus infection: Sudden onset of fever and flu-like symptoms. However, the course of DOBV infection resulted in a rapid decline of general condition and required admission to intensive care unit on day eight after onset of symptoms because of progressive respiratory problems with beginning hypoxia. The maximal and minimal levels of laboratory parameters differed between PUUV and DOBV-Sochi disease (Table 3). It is to note that the absolute peak and nadir levels probably occurred before admission. Thereby, the impairment of laboratory parameter levels may be underestimated particularly with regard to DOBV-Sochi infection because the admission occurred two days later compared to the PUUV-infected patient. We observed elevated levels of lipase and P-amylase in the patient infected with DOBV-Sochi, indicating a possible hantavirus-related acute pancreatitis. The association of HFRS with acute pancreatitis was described for several cases of infection with Dobrava-Belgrade and Hantaan virus [22–24], but not for infections with Puumala virus [25].

**Table 1:** Nucleotide (nt) and amino acid (aa) sequence identities (%) of partial DOBV-Sochi sequences

	S segment		M segment		L segment	
Virus isolate^a^	nt	aa	nt	aa	nt	aa
Sochi/hu	98.6	98.4	97.4	98.9	98.6	100.0
Sochi/Ap	98.8	98.9	97.4	98.9	n.a.^b^	n.a.
10645/Ap	98.6	98.9	n.a.	n.a.	99.4	100.0

^a^Sequences were amplified from serum sample of our DOBV-Sochi (2014) infected patient and compared to published sequences of DOBV-Sochi strains isolated from human (hu) and *Apodemus ponticus* (Ap). Accession numbers for nucleotide and amino acid sequences: Sochi/hu (S, M, L segment): JF920150, JF920149, JF920148 and AES92929, AES92928, AES92927; Sochi/Ap (S, M segment): EU188449, EU188450 and ABY64966, ABY64967; 10645/Ap (S, L segment): KP878312, KP878309 and ALP44173, ALP44170

^b^ *n.a* not available

**Table 2:** Characteristics and symptoms of two patients infected with PUUV and DOBV-Sochi

	PUUV	DOBV-Sochi
Age (years)	25	20
Body weight change (kg)	0.8	10
BMI^a^ at discharge	18.4	18.5
Hospitalization (days)	9	18
Intensive care unit stay (days)	0	2
Maximum temperature (°C)	39.6	40.0
Headache	n.d.^b^	+
Abdominal pain	+	+
Back-/side pain	+	-
Myalgia	n.d.	+
Pain in the limbs	n.d.	+
Nausea	+	+
Vomiting	+	+
Diarrhea	-	+
Obstipation	+	-
Night sweats	-	-
Dyspnea	-	+
Cough	+	-
Pleural effusion	-	+
Pulmonary congestion	-	+
Pulmonary edema	-	-
Infiltrates	-	-
Vertigo	+	-
Petechiae	-	+
Edema	-	+
Ascites	+	-
Hyperkalemia	+	+
Dialysis (number)	+ (1)	+ (6)

^a^ *BMI* body mass index, ^b^ *n.d* not determined

**Table 3:** Maximum and minimum levels of laboratory parameters of two patients with hantavirus infection

	PUUV	DOBV-Sochi	Reference values
Serum creatinine (mg/dL)	10.89	9.34	0.1–1.3
Urea (mg/dL)	120	231	<45
Uric acid (mg/dL)	6.5	11	<6
Serum albumin (g/L)	31.1	27.2	30–50
CRP (mg/L)^a^	61.2	101.5	<5
LDH (U/L)^b^	422	553	<248
Lipase (U/L)	24	860	<51
P-amylase (U/L)	25	520	8–53
Bilirubin total (mg/dL)	0.9	0.9	<1.0
GPT (U/L)^c^	59	55	<35
GOT (U/L)^d^	66	87	<35
γ-GT (U/L)^e^	77	81	<40
Alkaline phosphatase (U/L)	90	129	55-105
Hemoglobin (g/dL)	11	8.2	12–15
Hematocrit (L/L)	0.31	0.25	0.36–0.47
Platelets (10^9^/L)	51	53	150–440
Leukocytes (10^9^/L)	7.57	13.07	4–10

^a^ *CRP* C-reactive protein, ^b^ *LDH* lactate dehydrogenase, ^c^ *GPT* glutamate pyruvate transaminase, ^d^ *GOT* glutamate oxalacetate transaminase, ^e^ *γ-GT* γ -glutamyl transferase

**Fig. 1:**
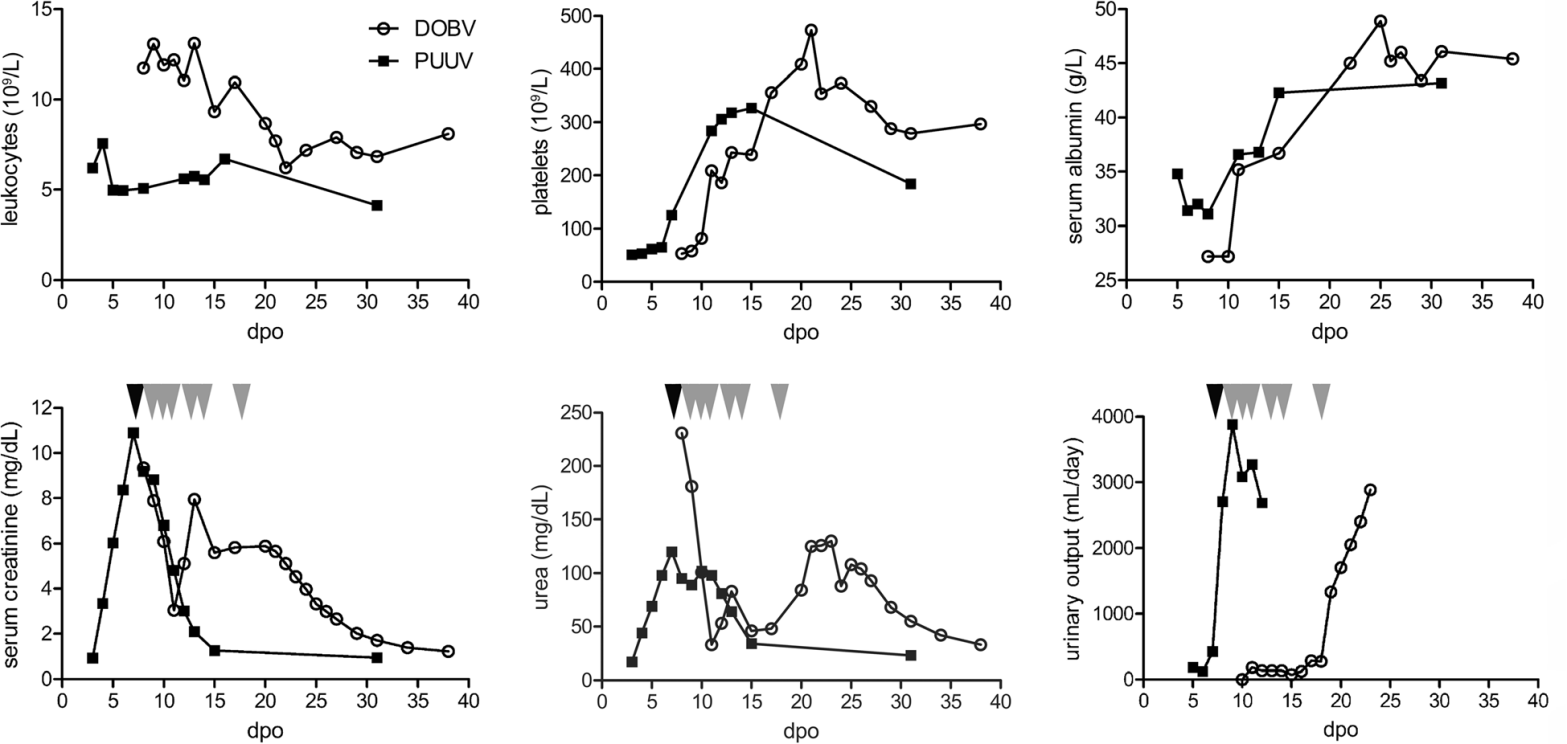
Course of laboratory parameters in patients infected with DOBV-Sochi and PUUV. *Black* and *gray* arrowheads indicate dialysis in PUUV and DOBV-Sochi patient, respectively. dpo, days post onset

**Fig. 2:**
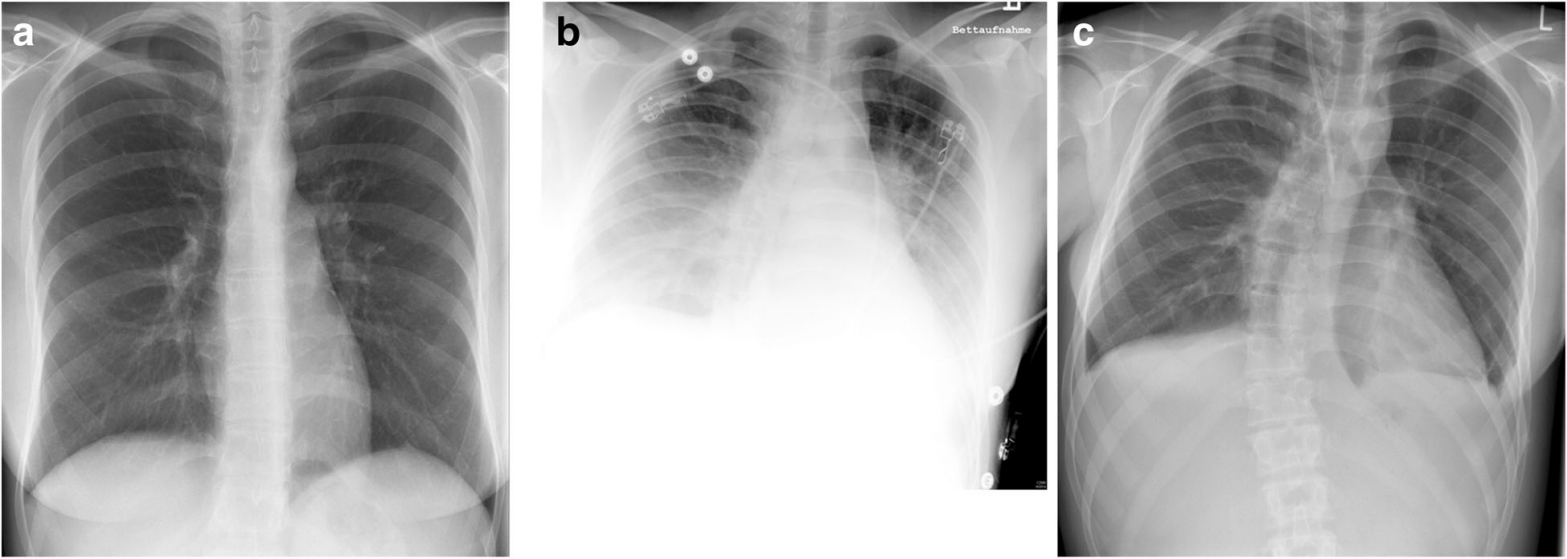
Chest x-ray of patients infected with PUUV (**a**, admission) and DOBV-Sochi (**b**, admission, bedside chest x-ray; **c**, after renal replacement therapy, 12 dpo)

Urine analysis revealed proteinuria and the presence of erythrocytes and leukocytes in the urine with higher cell counts for erythrocytes (43 cells/μl versus 561 cells/μl) and leukocytes (6 cells/μl versus 34 cells/μl) in the patient with DOBV-Sochi. Apart from these characteristic urine pathologies, both patients developed uremia and oliguria. Glucosuria, pollakiuria, nycturia or dysuria were not observed. Lastly, they suffered from anuria in the further clinical course. As a consequence, renal replacement therapies were applied. The reasons for dialysis were uremia and severe fluid overload for DOBV-Sochi patient and uremia for PUUV patient. The patient infected with PUUV infection was dialyzed once on day seven after onset of symptoms, whereas the patient with DOBV-Sochi infection underwent dialysis six times between day nine and day 18 after onset of symptoms (Fig. 1). With exception of scleral bleeding and petechiae in the patient with DOBV-Sochi infection, no bleedings, such as epistaxis, hematoma, melena or hematochezia, were observed in the two patients. Symptoms of involvement of the respiratory tract were cough in the case of PUUV infection, pleural effusion and pulmonary congestion in the DOBV-Sochi patient (Fig. 2). The patient with DOBV-Sochi presented with tachycardia. No other cardiovascular or other extrarenal organ manifestations were observed. Patients did neither exhibit ophthalmological symptoms nor complications of the CNS.

The analysis of the course of laboratory parameters in DOBV-Sochi infection demonstrated a prolonged phase with elevated levels of leukocytes and serum creatinine and decreased levels of thrombocytes and serum albumin compared to infection with PUUV (Fig. 1). Several parameters, e.g. thrombocytopenia, have been described to be associated and predictive for severe courses of hantavirus disease [26–28]. A low platelet count (<60 × 10^9^/L) indicates a subsequent acute renal failure with a rise in serum creatinine levels in Puumala virus infection [27, 29]. Corresponding to this definition for severe cases of PUUV infection, we observed platelet level of 51 × 10^9^/L for the patient with PUUV infection. For the DOBV-Sochi patient the level (53 × 10^9^/L) was also below 60 × 10^9^/L on admission.

The hospitalization of the patient with PUUV infection lasted nine days, whereas the patient with DOBV-Sochi infection was hospitalized for 18 days. The outcome of the hantavirus infection of both patients was complete recovery of renal function.

**Fig. 3:**
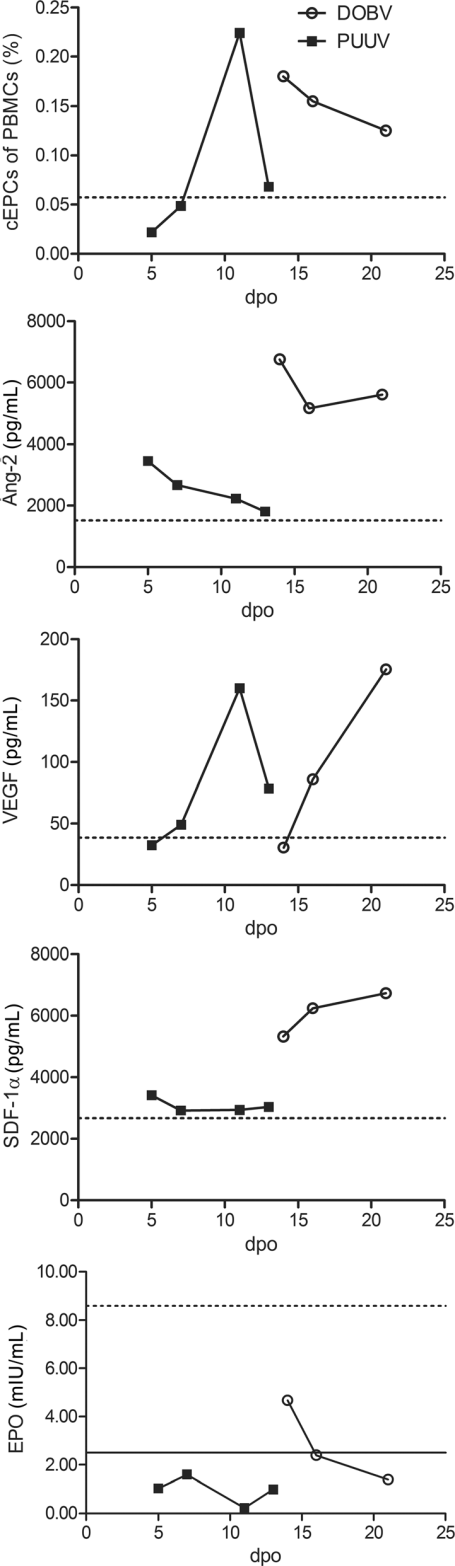
Course of cEPC numbers and plasma cytokine levels during hantavirus infection with DOBV-Sochi and PUUV. Horizontal dashed lines indicate the mean levels of 23 healthy control persons. EPO levels of some patient samples were below the limit of detection of the assay (<2.5 mIU/ml, horizontal line)

Our previous studies revealed the role of circulating endothelial progenitor cells (cEPCs) and cEPCs-mobilizing cytokines in the clinical course of patients infected with PUUV [30]. As the normalization of laboratory parameters is paralleled to the mobilization of cEPCs, we analyzed the levels of cEPCs and of cEPC-mobilizing cytokines in the patients (Fig. 3). Quantification of levels of cEPCs by flow cytometry and of cytokines by Quantikine enzyme-linked immunosorbent assay (ELISA; R&D Systems) of patients and of 23 healthy persons was performed as described previously [30].

Both patients demonstrated an increase in levels of cEPCs, Ang-2, VEGF, and SDF-1α compared to levels observed in healthy controls. Erythropoietin (EPO) levels were decreased during the disease indicating damage to the EPO-producing renal cells. All four samples of the PUUV patient and the samples of day 16 and 21 of the DOBV-Sochi patient were below detection limit of the EPO assay (<2.5 mIU/ml). Besides the varying extent of cytokine level elevation, differences existed in the course of cEPC and cytokine level changes between both infections. A prolonged elevation of cEPC levels with a slow normalization in the patient with DOBV-Sochi infection was observed. The duration of the increase of Ang-2 and SDF-1α levels was also extended and much higher in DOBV-Sochi infection than in infection with PUUV. Furthermore, levels of VEGF in DOBV-Sochi infection increased later than in PUUV infection. The same delay was observed for the decrease of EPO levels. Taken together, both infections are characterized by mobilization of cEPCs and cytokine level elevation, but the temporal course and the extent of increase of cytokine levels differ enormously between infection with PUUV and DOBV-Sochi.

## Conclusions

Among Old World hantaviruses, DOBV genotype Sochi is characterized by severe clinical course and a high CFR of 14.5% [13]. In contrast, PUUV disease exhibits a low CFR of less than 1% [31]. However, diseases caused by DOBV-Sochi and PUUV infection may also differ individually and cases of severe hantavirus disease due to PUUV infection were reported [14, 32, 33]. We compared two severe hantavirus infections caused by these two different virus species, PUUV and DOBV-Sochi. The two cases did not differ significantly with regard to symptoms and organ involvement. However, the DOBV-Sochi infection presented with an enhanced impairment of laboratory parameters and a prolonged renal phase. The more severe clinical picture of infection with DOBV compared to PUUV corresponds to the observations made in other studies that compared infections with PUUV and different DOBV genotypes. Patients with DOBV infections were more often hypotensive, exhibited higher levels of serum creatinine, displayed more severe thrombocytopenia and required dialysis more often compared to patients infected with PUUV [34–36]. Our analysis of the clinical course of the two infections revealed further differences between the two infections. Impairment of laboratory parameters and upregulation of cytokines were prolonged in DOBV-Sochi infection. The mechanisms that are responsible for the more severe and protracted course of DOBV-Sochi infection are not completely understood. Previous studies have demonstrated a role of endothelial activation and repair in the clinical course of PUUV infection [30, 37]. The normalization of clinical parameters has been paralleled to the mobilization of endothelial progenitor cells. We also observed a mobilization of cEPCs in these two hantavirus infected patients. Similar to the observations for PUUV infection, levels of cEPCs and mobilizing cytokines were elevated in DOBV-Sochi infections. However, the increase of cytokines started later after onset of symptoms and was higher in infection with DOBV-Sochi than in PUUV infection.

Different cytokines were discussed to be responsible for hantavirus pathogenesis [9, 38–41]. Several studies analyzed the role of VEGF in hantavirus disease [30, 42–45]. The effect of VEGF seems to be temporally regulated. Early and localized upregulation of VEGF may be responsible for the clinical symptoms such as capillary leakage during hantavirus infection. In contrast, late and systemic elevation of VEGF may contribute to endothelial repair. As shown for VEGF, Ang-2 may also contribute to the pathogenesis of hantavirus disease. Altered ratios between angiopoietin-1 and −2 impair the barrier function of the endothelial monolayer during Dengue virus infection [46]. The levels of Ang-2 were much higher in the patient with DOBV-Sochi infection than in the one with PUUV.

A reason for the altered clinical course and cytokine deregulation of DOBV-Sochi infection may be the enhanced replication of the virus. Viral load and antibody response influence the severity and the clinical course of hantavirus infection [47–49]. Unfortunately, we could neither measure the titer of hantaviral genomes nor of hantavirus-specific antibodies to explore a possible association between clinical course and viral titer or antibody response in our patients. It would be of interest to analyze if the differences observed in these two cases are specific for DOBV-Sochi infections compared to PUUV infections in a larger cohort of patients. The comparison of the clinical course of hantavirus genotypes with different pathogenicity may help to explore the underlying mechanisms. It seems that the infections were very similar in symptoms and induce the same pathways of cytokine signaling and endothelial damage and repair. However, research should further focus on the observed differences in the kinetics of cytokine mobilization. As observed for VEGF in hantavirus infection, cytokines may have detrimental as well as beneficial effects during the clinical course. Therefore, the knowledge about the role of cytokines in the clinical course and its temporospatial regulation in infections with different pathogenic hantavirus is crucial for the development of therapeutic strategies interfering with cytokine signaling.

The comparison of hantavirus disease caused by infection with PUUV and DOBV-Sochi revealed a more severe course for DOBV-Sochi. Initial symptoms and organ involvement did not vary noticeably. The two infections differed especially in the course and levels of cytokine upregulation. These results may indicate that temporal control and high level upregulation of certain cytokines contribute to the severity of the clinical course of hantavirus disease.

## Abbreviations

Ang-2: angiopoietin-2
AV: atrioventricular
BMI: body mass index
cEPC: circulating endothelial progenitor cells
CFRs: case fatality rates
CRP: C-reactive protein
DOBV: Dobrava-Belgrade virus
dpo: days post onset
EPO: erythropoietin
GOT: glutamate oxalacetate transaminase
GPT: glutamate pyruvate transaminase
HCPS: hantaviral cardiopulmonary syndrome
HFRS: hemorrhagic fever with renal syndrome
HTNV: Hantaan virus
LDH: lactate dehydrogenase
PUUV: Puumala virus
SDF-1α: stromal derived factor-1α
VEGF: vascular endothelial growth factor
γ-GT: γ -glutamyl transferase
